# Effect of Miscellaneous Meals Replacing Soybean Meal in Feed on Growth Performance, Serum Biochemical Parameters, and Microbiota Composition of 25–50 kg Growing Pigs

**DOI:** 10.3390/ani14091354

**Published:** 2024-04-30

**Authors:** Xianliang Zhan, Lei Hou, Zhentao He, Shuting Cao, Xiaolu Wen, Shuai Liu, Yaojie Li, Shaozhen Chen, Huayu Zheng, Dongyan Deng, Kaiguo Gao, Xuefen Yang, Zongyong Jiang, Li Wang

**Affiliations:** State Key Laboratory of Livestock and Poultry Breeding, Key Laboratory of Animal Nutrition and Feed Science in South China, Ministry of Agriculture and Rural Affairs, Guangdong Provincial Key Laboratory of Animal Breeding and Nutrition, Maoming Branch, Guangdong Laboratory for Lingnan Modern Agriculture, Institute of Animal Science, Guangdong Academy of Agricultural Sciences, Guangzhou 510640, China; zhanxianliang1996@163.com (X.Z.); rhoulei@126.com (L.H.); kuzma022133@gmail.com (Z.H.); wenxiaolu@gdaas.cn (X.W.); 15027016245@163.com (S.L.); lyj1714659617@163.com (Y.L.); 13413566063@163.com (S.C.); 17872305992@163.com (H.Z.); 15675858457@163.com (D.D.); gaokaiguo312@126.com (K.G.); yangxuefen@gdaas.cn (X.Y.); jiangzy@gdaas.cn (Z.J.)

**Keywords:** miscellaneous meals, soybean meal, growth performance, microbiota

## Abstract

**Simple Summary:**

Given the escalating production of livestock and poultry, coupled with the surging cost of soybean meal, the search for alternative raw materials that can replace soybean meal is becoming more important. Agricultural by-products like rapeseed meal, cottonseed meal, and sunflower seed meal present a promising plant-based protein alternative to soybean meal in pig production. This study revealed that corn–soybean–miscellaneous meals and corn–miscellaneous meals significantly enhanced the average daily growth of pigs weighing 25–50 kg compared to a corn–soybean meal diet. These findings suggest that miscellaneous meals can effectively serve as an alternative feed ingredient to soybean meal in pig diets. This research can be helpful to further develop miscellaneous meals (rapeseed meal, cottonseed meal, and sunflower meal) as a functional alternative feed ingredient to soybean meal.

**Abstract:**

The present study aims to determine the effect of miscellaneous meals (rapeseed meal, cottonseed meal, and sunflower meal) replacing soybean meal in feed on growth performance, apparent digestibility of nutrients, serum biochemical parameters, serum free amino acid content, microbiota composition and SCFAs content in growing pigs (25–50 kg). A total of 72 (Duroc × Landrace × Yorkshire) growing pigs with initial weights of 25.79 ± 0.23 kg were randomly divided into three treatments. The pigs were fed corn–soybean meal (CON), corn–soybean–miscellaneous meals (CSM), and corn–miscellaneous meals (CMM). Each treatment included six replicates with four pigs per pen (n = 24, 12 barrows and 12 gilts). Soybean meal accounted for 22.10% of the basal diet in the CON group. In the CSM group, miscellaneous meals partially replaced soybean meal with a mixture of 4.50% rapeseed meal, 3.98% cottonseed meal, and 4.50% sunflower meal. In the CMM group, miscellaneous meals entirely replaced soybean meal with a mixture of 8.50% rapeseed meal, 8.62% cottonseed meal, and 8.5% sunflower. The results showed that compared with the CON, the CSM and CMM groups significantly improved the average daily gain (ADG) of growing pigs during the 25–50 kg stage (*p* < 0.05) but had no effects on average daily feed intake (ADFI) and average daily feed intake/average daily gain (F/G) (*p* > 0.05). Moreover, the CMM group significantly reduced nutrient apparent digestibility of gross energy compared with the CON group. The serum biochemical parameters results showed that the CSM group significantly improved the contents of total protein (TP) compared with the CON group (*p* < 0.05). The CMM group significantly improved the contents of total protein (TP), high-density lipoprotein cholesterol (HDL-C), and low-density lipoprotein cholesterol (LDL-C) compared with the CON group in serum (*p* < 0.05). In comparison with the CON group, the CMM group also significantly improved lysine (Lys), threonine (Thr), valine (Val), isoleucine (Ile), leucine (Leu), phenylalanine (Phe), arginine (Arg), and citrulline (Cit) levels in serum (*p* < 0.05). However, the CMM group significantly decreased non-essential amino acid content glycine (Gly) in serum compared with CON (*p* < 0.05), while compared with the CON group, the CSM and CMM groups had no significant effects on the relative abundance, the alpha-diversity, or the beta-diversity of fecal microbiota. Moreover, compared with the CON group, the CSM group significantly increased butyric acid and valeric acid contents of short-chain fatty acids (SCFAs) in feces (*p* < 0.05). In contrast to the CON group, the CMM group significantly reduced the contents of SCFAs in feces, including acetic acid, propionic acid, and isobutyric acid (*p* < 0.05). Collectively, the results of the present study indicate that miscellaneous meals (rapeseed meal, cottonseed meal, and sunflower meal) can partially replace the soybean meal and significantly improve the growth performance of growing pigs during the 25–50 kg stage. Thus, miscellaneous meals are a suitable protein source as basal diets to replace soybean meals for 25–50 kg growing pigs. These results can be helpful to further develop miscellaneous meals as a functional alternative feed ingredient to soybean meal.

## 1. Introduction

Soybean meal is a steady protein source that is most widely used in swine feed [1]. Soybean meal has high digestibility, consistent processing methods, and an excellent amino acid profile [2]. The burgeoning sectors of livestock, poultry, and aquaculture have catalyzed a pronounced escalation in soybean meal demand, a trend paralleled by a swift ascent in market prices. This scenario accentuates the critical imperative for the identification and integration of novel protein raw materials within pig diets, a pursuit that is gaining momentum as a strategic priority in contemporary animal agriculture.

Rapeseed meal is a co-product of oil and biofuel production [3]. Research indicates that incorporating rapeseed meal and fava beans into the diets of growing pigs (108.7 ± 4.2 kg final body weight) enhances feed conversion rates during the fattening period and elevates blood levels of free amino acids [4]. At the same time, the high fiber level of rapeseed meal reduces the ileal digestibility of pigs (initial live weight of 42.1 ± 3.0 kg) [5]. Concurrently, cottonseed and sunflower meals have emerged as promising soybean meal alternatives in pig nutrition, drawing increasing scholarly attention [6,7,8]. Cottonseed meal has the advantages of low price and high crude protein content when it is used as a protein raw material in animal feed production, although the high content of free gossypol in cottonseed meal seriously restricts its use proportion [9,10]. Sunflower meal is a by-product of sunflower seeds after pre-pressing or direct extraction. Sunflower meal is more easily digested and absorbed by animals, with a protein content generally ranging from 27.4% to 37.0% [11,12]. Given the disparities in price, availability, and nutritional profiles, the strategic combination of these diverse meal sources presents a viable pathway toward reducing the reliance on soybean meal in pig diets.

Recent studies by our team have shown that replacing soybean meal with miscellaneous meals (rapeseed meal, cottonseed meal, and sunflower seed meal) in the diets of 50–75 kg growing and finishing pigs does not significantly impact their growth performance, nutrient digestibility, serum biochemical parameters, or microbiota diversity [13]. These observations indicate the feasibility of employing miscellaneous meals (rapeseed meal, cottonseed meal, and sunflower seed meal) for replacing soybean meal in pig diets, without detrimentally affecting health or growth performance. However, it is unclear whether replacing soybean meal with miscellaneous meals (rapeseed meal, cottonseed meal, and sunflower meal) has effects on normal diets for 25–50 kg growing pigs. Thus, in the present study, we used miscellaneous meals (rapeseed meal, cottonseed meal, and sunflower meal) to partly or entirely replace soybean meal in feed to explore the effects on growth performance, apparent digestibility of nutrients, serum biochemical parameters, free amino acid contents, fecal microbiota, and short-chain fatty acid (SCFA) contents in growing pigs during the 25–50 kg stage.

## 2. Materials and Methods

### 2.1. Animals, Diets, and Management

Animal protocols in the present study were performed following the Guidelines for the Care and Use of Animals for Research and Teaching following approval by the Animal Care and Use Committee of Guangdong Academy of Agricultural Science (authorization number GAASIAS-2016-017).

A total of 72 (Duroc × Landrace × Yorkshire) growing pigs with an initial weight of 25.79 ± 0.23 kg were randomly divided into three treatments. All 18 pens were identical, with the same covered area (3 m^2^/pig) and were equipped with similar troughs for feed concentrates and water. Pigs were provided ad libitum access to water and feed during the entire experimental trial. Pigs were fed corn–soybean meal (CON), corn–soybean–miscellaneous meals (CSM), and corn–miscellaneous meals (CMM). Each treatment included six replicates with four pigs per pen (n = 24, 12 barrows and 12 gilts). Soybean meal accounted for 22.1% of basal diet in CON. In the CSM group, miscellaneous meals replaced soybean meal partly with 4.5% rapeseed meal, 3.98% cottonseed meal, and 4.5% sunflower meal. Miscellaneous meals replaced soybean meal entirely with 8.5% rapeseed meal, 8.62% cottonseed meal, and 8.5% sunflower meal in the CMM group. In addition, three groups had the same protein levels at 16% in experimental diets. The CSM and CMM groups were given additional crystalline amino acids to ensure that the standardized ileal digestibility amino acids level was the same as the CON. The dietary preparation of experimental animals was formulated to meet or exceed the National Research Council recommendations from 2012. All pigs had free access to feeding and drinking water. The composition and nutrient contents of the experimental diets are listed in Table 1. The nutrient information for rapeseed meal, cottonseed meal, and sunflower seed meal is presented in Table 2.

### 2.2. Growth Performance

The average daily feed intake (ADFI) was calculated by recording daily feed consumption. The average daily gain (ADG) of piglets was calculated based on their weight obtained at the beginning and end of the experiment. The feed-to-gain ratio (F/G) was calculated based on the feed intake and weight gain throughout the experiment period. The equations utilized for the determination of growth performance metrics are specified below:

ADG = (Final body weight − Initial body weight)/days of feeding;

ADFI = (total feed consumed − residual feed)/(duration of feeding period × number of pigs);

F/G = ADFI/ADG.

### 2.3. Apparent Digestibility of Nutrients

All diets were given a starting dose of titanium dioxide (0.4%), which was used as an indigestible marker of nutrient apparent digestibility. In the pens, feces were collected, dried, sampled, and stored at −20 °C for analysis. All of the growing pigs’ excrement was defrosted, blended, and then baked at 65 °C for 72 h before the natural moisture was restored at room temperature for 24 h. The crude protein content was estimated by multiplying the total nitrogen content measured by the Kjeltec 8400 analyzer (FOSS Analytical AB, Höganäs, Sweden) by a coefficient of 6.25. The crude fat content was measured using an automatic extraction analyzer (XT 15i, Ankom Technology, Rochester, NY, USA). The total energy content in the diet was determined by an oxygen bomb calorimeter (6400, Parr Instrument, Moline, IL, USA) according to the international standard ISO 9831:1998 [14] method. Nutrient apparent digestibility was calculated as follows: apparent nutrient digestibility (%) = [1 − (TiO_2_ content in the dietary/TiO_2_ content in the fecal sample) × (nutrient content in the fecal sample/nutrient content in the dietary)] × 100 [13]. The nutrient values of rapeseed meal, cottonseed meal, and sunflower seed meal were measured according to the methods above.

### 2.4. Serum Biochemical Parameters

On the last day of the experiment, the feed was cut off at 8:00 p.m. At 8:00 a.m. the next day morning, one pig was selected from each column for blood collection. A total of 10 mL of blood was collected from the veins at the ear margin of the pig, centrifuged at 3500 r/min for 10 min to separate the serum, and sub-packed in sterile centrifuge tubes. Serum samples were received and stored at −80 °C for the determination of serum biochemical parameters and free amino acid contents. During the index analysis, the serum was positioned in an ice water bath to preserve its activity. The contents of the total protein (TP), creatinine (CRE), aspartate aminotransferase (AST), alkaline phosphatase (ALP), alanine aminotransferase (ALT), albumin (ALB), urea (UREA), glucose (GLU), triglyceride (TG), cholesterol (CHO), high-density lipoprotein cholesterol (HDL-C), and low-density lipoprotein cholesterol (LDL-C) (BioSino Bio-Technology & Science Inc., Beijing, China) in serum were measured by an automatic biochemical analyzer (Selectra Pro XL, Vital Scientific, Spankeren, Gelderland, The Netherlands) according to the manufacturer’s instructions. All serum biochemical kits used were purchased from Zhongsheng Beikong Biotechnology Co., Beijing, China.

### 2.5. Serum Free Amino Acid Contents

A total of 0.4 mL of the serum sample was accurately sucked into a sterile centrifuge tube. We added 1.2 mL of 10% sulfur sodium salicylate to precipitate the sample protein, shook it well, and centrifuged at 4 °C at 12,000 r/min for 15 min. Afterward, we took the supernatant and used a 0.22 μ filter for the water phase filter membrane and put it on the machine. The concentration of free amino acids in serum was determined by using an automatic amino acid analyzer (L-8900, Hitachi, Ibarakiken, Japan) based on the principle of the Ninhydrin post-column derivation method.

### 2.6. Feces Microbiota

The total genomic DNA of the feces digested samples were extracted using a QIAamp DNA kit (Qiagen, Hilden, Germany) and DNA quality was evaluated by electrophoresis on 1% agarose gels. The V3-V4 region of 16S rRNA was amplified by PCR using specific barcode primers for all of the colonic digesta samples. A total volume of 30 µL was used in the PCR reactions and the amplification products were purified using an Ion Plus Fragment Library Kit 48 rxns (Thermo Scientific, Waltham, MA, USA) to construct the library and using a Qubit 2.0 Fluorometer (Thermo Scientific) to detect the quality of the library. After that, the low-quality part of the reads was cut by Cutadapt (V1.9.1). Next, the sample data from the reads, obtained by barcode, cut off the barcode, the primer sequence, and the raw data (raw reads) after the above processing. Furthermore, the sequence of reads obtained was compared with the species annotation database to remove the chimera sequence to establish valid data (clean reads). Operational taxonomic units (OTUs) were delineated from the clean reads using Uparse software (version 7.0.1001). To assess microbial community diversity within samples (alpha diversity) and between samples (beta diversity), analyses were conducted utilizing Qiime software (version 1.9.1). Alpha diversity metrics included the Observed_species, Shannon, Simpson, Chao1, Ace, and PD_whole_tree indices. Principal component analysis (PCA), principal coordinate analysis (PCoA), and non-metric multidimensional scaling (NMDS) were performed to calculate the β diversity between groups.

### 2.7. SCFA Content

The contents of short-chain fatty acids (SCFAs) in feces were determined using a liquid chromatography mass spectrometer (Agilent Technologies, Santa Clara, CA, USA). Briefly, 0.1 g of colonic digesta sample was mixed with 1 mL of 5 mmol NaOH and 50 mL of 0.5 μg mL^−1^ hexanoic acid 3. The supernatant was collected after centrifuging at 12,000× *rpm*, 4 °C for 15 min. Then, the mixture (including the above supernatant, 250 μL of propanol/pyridine (*v*/*v*, 3:2), 150 μL of water, and 50 μL of propyl chloroformate) was mixed into derivatives for 5 min and extracted twice with n-hexane. A 10 mg anhydrous Na_2_SO_4_ was used for dehydration, and 150 mg supernatant was collected for analysis.

### 2.8. Statistical Analysis

Data were analyzed using SPSS 20.0 (SPSS Inc., Chicago, IL, USA) and are presented as the mean ± standard error (SEM). The results were assessed by one-way analysis of variance (ANOVA) followed by Tukey’s test. Differences were considered significant at *p* < 0.05.

## 3. Results

### 3.1. Growth Performance

The results in the present study showed that, compared with the CON group, the CSM group significantly improved the ADG of growing pigs during the 25–50 kg (*p* < 0.05) stage but had no effects on the ADFI and F/G (*p* > 0.05). Concurrently, the CMM group also significantly improved the ADG of growing pigs (*p* < 0.05) but had no effects on the ADFI and F/G (*p* > 0.05) (Table 3).

### 3.2. Apparent Digestibility of Nutrients

As shown in Table 4, compared with the CON group, the CSM group had no significant effects on the nutrient apparent digestibility, including crude protein, ether extract, and gross energy (*p* < 0.05). However, the CMM group significantly reduced nutrient apparent digestibility of gross energy in growing pigs during the 25–50 kg stage compared with the CON group (*p* < 0.05).

### 3.3. Serum Biochemical Parameters

Based on the findings in Table 5, compared with the CON group, the CSM group significantly improved the contents of TP in the serum of growing pigs during the 25–50 kg (*p* < 0.05) stage, along with no significant effects on ALT, CRE, AST, ALP, ALB, UREA, GLU, TG, CHO, HDL-C, or LDL-C. In addition, compared with the CON group, the CMM group significantly improved the contents of TP, HDL-C, and LDL-C in serum (*p* < 0.05), but not affect the contents of ALT, CRE, AST, ALP, ALB, UREA, GLU, TG, CHO in serum.

### 3.4. Serum Free Amino Acid Contents

In the present study, our results indicate that the CSM group had no significant effects on the contents of free amino acid in serum compared with the CON group. The CMM group significantly improved essential amino acids (Lys, Thr, Val, Ile, Leu, Phe, Arg), non-protein amino acids, and derivatives/metabolites of amino acids (Cit), and significantly decreased non-essential amino acids contents (Gly) compared with the CON group (*p* < 0.05) (Table 6).

### 3.5. Microbial Composition of Feces

**Figure 1:**
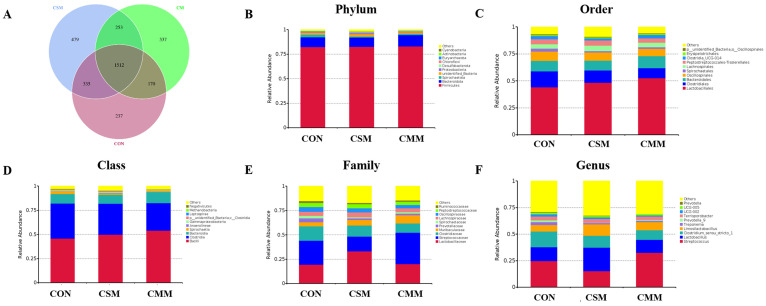
The Venn diagram and relative abundances of bacteria at the phylum, class, order, family, and genus levels. (**A**) Venn diagram. (**B**) Top 10 bacteria at the phylum level. (**C**) Top 10 bacteria at the class level. (**D**) Top 10 bacteria at the order level. (**E**) Top 10 bacteria at the family level. (**F**) Top 10 bacteria at the genus level. Abbreviations: CON, corn–soybean meal; CSM, corn–soybean–miscellaneous meal; CMM, corn–miscellaneous meal.

The results of the 16s RNA sequencing of fecal microbes show that there were 1512 OTUs among the three groups (Figure 1A); Figure 1 also shows the microbial composition at the phylum (Figure 1B), order (Figure 1C), class (Figure 1D), family (Figure 1E), and genus (Figure 1F) levels. Furthermore, at the phylum level, the 10 most prevalent microbes were *Firmicutes*, *Bacteroidota*, *Spirochaetota*, *unidentified Bacteria*, *Proteobacteria*, *Desulfobacterota*, *Chloroflexi*, *Euryarchaeota*, *Actinobacteria*, and *Cyanobacteria*. At the order level, the 10 most prevalent microbes were *Lactobacillales*, *Clostridiales*, *Bacteroidales*, *Oscillospirales*, *Spirochaetales*, *Lachnospirales*, *Peptostreptococcales-Tissierellales*, *Clostridia_UCG-014*, *Erysipelotrichales*, *unidentified_Bacteria*, and *Oscillospirales.* At the class level, the 10 most prevalent microbes were *Bacilli*, *Clostridia*, *Bacteroidia*, *Spirochaetia, Gammaproteobacteria*, *unidentified Bacteria*, *Clostridia*, *Methanobacteria*, *Anaerolineae*, *Negativicutes*, *Alphaproteobacteria.* At the family level, the 10 most prevalent microbes were *Lactobacillaceae*, *Streptococcaceae*, *Clostridiaceae*, *Muribaculaceae*, *Prevotellaceae*, *Spirochaetaceae*, *Lachnospiraceae*, *Oscillospiraceae*, *Peptostreptococcaceae*, *Ruminococcaceae.* At the genus level, the 10 most prevalent microbes were *Streptococcus*, *Lactobacillus*, *Clostridium-sensu-stricto-1*, *Limosilactobacillus*, *Treponema*, *Prevotella-9*, *Terrisporobacter*, *UCG-002*, *UCG-005*, and *Prevotella*.

**Figure 2:**
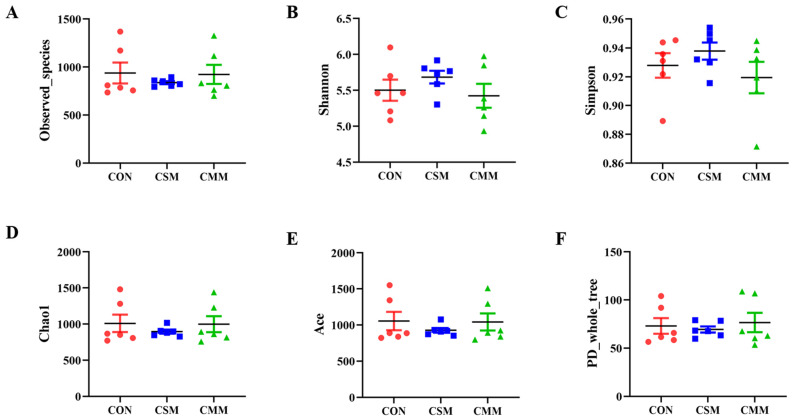
Effect of miscellaneous meals (rapeseed meal, cottonseed meal, and sunflower seed meal) replacing soybean meal partly/entirely in feed on the alpha diversity of fecal microbial in 25–50 kg growing pigs. (**A**) Observed_species index. (**B**) Shannon index. (**C**) Simpson index. (**D**) Chao1 index. (**E**) Ace index. (**F**) PD_whole_tree index. Abbreviations: CON, corn–soybean meal; CSM, corn–soybean–miscellaneous meal; CMM, corn–miscellaneous meal.

As shown in Figure 2, the replacement of soybean meal with a miscellaneous meal (rapeseed meal, cottonseed meal, and sunflower seed meal) in the diet did not significantly impact the alpha diversity of fecal microbiota, as indicated by the Observed species index, Shannon index, Simpson index, Chao1 index, Ace index, and PD_whole_tree index (*p* > 0.05).

**Figure 3:**
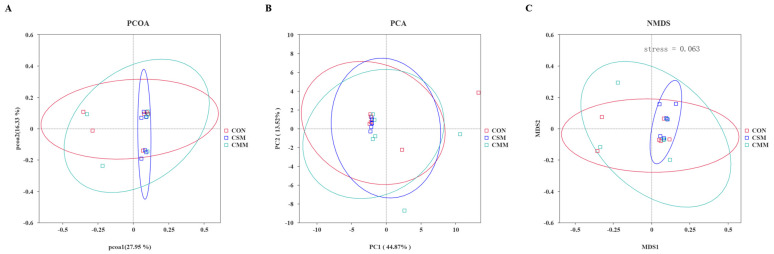
Effect of miscellaneous meals (rapeseed meal, cottonseed meal, and sunflower seed meal) replacing soybean meal partly/entirely in feed on the beta diversity of fecal microbial in 25-50 kg growing pigs. (**A**) Principal coordinate analysis (PCoA) plots. (**B**) Principal component analysis (PCA). (**C**) The non-metric multidimensional scaling (NMDS). Abbreviations: CON, corn–soybean meal; CSM, corn–soybean–miscellaneous meal; CMM, corn–miscellaneous meal.

Regarding beta-diversity, the PCoA, PCA, and NMDS analyses demonstrated that the distributional distances among the three groups (CON, CSM, and CMM) did not show a clear separation (Figure 3).

### 3.6. SCFAs Content

The present results indicated that the CSM group significantly increased butyric acid and valeric acid contents of SCFAs compared with the CON group in feces (*p* < 0.05). The CMM group significantly reduced the contents of SCFAs in feces, including acetic acid, propionic acid, and isobutyric acid, compared with the CON group (*p* < 0.05) (Table 7).

## 4. Discussion

The traditional feed formula for animal husbandry is mainly a corn–soybean meal type, which requires a large amount of soybean meal consumption. Recent trends showcase a persistent escalation in the cost of primary feed ingredients, such as soybeans, precipitating a marked augmentation in feed expenditure. Soybean meal is a by-product obtained from soybean oil extraction, with a high protein content of 43% to 47%, and is also rich in lysine and methionine, which are important for pigs [15,16]. In China, agricultural by-products such as rapeseed meal, cottonseed meal, and sunflower seed meal emerge as viable feed components, characterized by their substantial protein content, extensive production, and cost-effectiveness. These attributes position them as potential vegetable-based protein feedstocks capable of substituting soybean meal in pig diets. Recent studies by our team have shown that replacing soybean meal with miscellaneous meals (rapeseed meal, cottonseed meal, and sunflower seed meal) in the diets of 50–75 kg growing and finishing pigs does not significantly impact their growth performance, nutrient digestibility, serum biochemical parameters, or microbiota diversity [13]. However, it is not clear whether the replacement of soybean meal with miscellaneous meals has an effect on the early stages (25–50 kg) of growing pigs.

In our study, the results showed that miscellaneous meals (rapeseed meal, cottonseed meal, and sunflower meal) replacing soybean meal partly or entirely in feed significantly improved the ADG of growing pigs at 25–50 kg, but had no effects on ADFI or F/G. Velayudhan et al. found rapeseed meal, instead of a 30% soybean meal, in lactating sows’ diet had no negative effects on the reproductive performance of sows and the growth performance of their piglets [17]. The research results of Liu et al. indicate that high-protein rapeseed meal and traditional rapeseed meal can partially or completely replace soybean meal in the diet of pregnant and lactating sows, without negatively affecting the reproductive and litter performance of sows (initial body weight: 207.8 ± 29.11 kg) [18]. Rao et al. found that replacing soybean meal with sunflower seed meal in isocaloric and isonitrogenous diets did not influence the body weight gain or the relative weight of visceral organs in broilers [19]. Furthermore, a study has shown that growing pigs (initial body weight of 19.3 ± 1.8 kg) demonstrate enhanced digestive utilization efficiency with high-protein sunflower seed meal [20,21]. Our findings reveal that miscellaneous meals (rapeseed meal, cottonseed meal, and sunflower meal) significantly improve growth performance during the 25–50 kg stage of pigs. This research underscores the potential of these alternative feed ingredients to enhance the sustainability and cost-effectiveness of pig production by offering viable nutritional alternatives to traditional soybean meal-based diets.

In the realm of animal agriculture, the selection of appropriate feed ingredients is pivotal for enhancing the digestibility and utilization of feed nutrients, which in turn can mitigate the environmental footprint of animal production [16]. The present study indicated that miscellaneous meals (rapeseed meal, cottonseed meal, and sunflower meal) replacing soybean meal entirely, significantly reduced the gross energy digestibility of pigs, along with miscellaneous meals (rapeseed meal, cottonseed meal, and sunflower meal) replacing soybean meal partly had no significant effects on the nutrient apparent digestibility of growing pigs during the 25–50 kg stage. The study by Gu et al. reports that diets containing fermented cottonseed meal exhibited higher digestibility of several key nutrients (dry matter, organic matter, crude protein, and gross energy) compared to soybean meal diets at 14 days post-weaning [19]. Similarly, a study on the substitution of rapeseed meal showed that fecal nutrient digestibility had no significant difference between rapeseed meal and soybean meal diets during the growing period (13.18 to 39.81 kg) of pigs [22]. Furthermore, Liu et al. found that growing pigs (initial body weight of 22.68 ±0.18 kg) fed a rapeseed meal diet and a soybean meal diet showed no difference in the apparent total tract digestibility of energy [23]. Inconsistently, our results show that miscellaneous meals (rapeseed meal, cottonseed meal, and sunflower meal) replacing soybean meal in the diet significantly reduced the apparent total tract digestibility of energy in pigs during the 25–50 kg stage. This discrepancy underscores the complexity of feed formulation and its impact on nutrient digestibility, highlighting the need for further research to optimize dietary compositions that support sustainable and efficient animal production.

Serum biochemical parameters are widely recognized as critical indicators in nutritional assessment, offering insights into the physiological and metabolic conditions of animals [24]. In our study, miscellaneous meals (rapeseed meal, cottonseed meal, and sunflower meal) replacing soybean meal partly and entirely in feed significantly improved the content of total protein in serum compared with the CON group. TP levels are considered a reliable marker of the nutritional and metabolic health of animals [25]. Supporting this notion, Yang et al. showed that higher TP serum levels can promote tissue development in geese [26]. Furthermore, our study found that miscellaneous meals (rapeseed meal, cottonseed meal, and sunflower meal) replacing soybean meal entirely in feed significantly improved the contents of HDL-C and LDL-C. Recent research has illuminated the multifaceted roles of HDLs beyond cholesterol transport, including their antioxidant properties, stimulation of nitric oxide (NO) production, and their anti-inflammatory and anti-apoptotic effects [27,28]. These findings suggest that HDLs contribute to a range of beneficial physiological and metabolic processes. In general, the present study indicated that miscellaneous meals replacing soybean in diet had no negative effects on serum biochemical indicators in pigs during the 25–50 kg stage. On the contrary, our results indicate potential improvements in the nutritional and metabolic status of these animals, as evident by the increased TP and HDL-C alterations.

By the way, our study further revealed that compared with the CON group, miscellaneous meals (rapeseed meal, cottonseed meal, and sunflower meal) replacing soybean meal entirely in feed significantly improved the contents of serum free amino acids, including essential amino acids (Lys, Thr, Val, Ile, Leu, Phe, and Arg), non-protein amino acids, and derivatives/metabolites of amino acids (Cit), but significantly decreased non-essential amino acids contents (Gly). Amino acids play important roles in the body as key components of protein metabolism [29,30,31]. Complementary to our results, previous studies have shown that dietary supplementation with free amino acids in growing pigs (33.6 ± 0.65 kg) increases serum levels of essential amino acids such as Arg, His, Lys, Phe, Thr, Trp, and Val. This enhancement extends to non-essential amino acids, including Asp, Tyr, Glu, Gly, and Ser [32,33]. The observed enhancements in serum amino acid concentrations may be attributed to the addition of exogenous amino acids to the diet. The distinct protein and amino acid profiles inherent to soybean meal, rapeseed meal, cottonseed meal, and sunflower meal necessitated the supplementation of amino acids in the diets of the miscellaneous meal (rapeseed meal, cottonseed meal, and sunflower meal) groups to maintain equivalent protein and amino acid levels across all dietary treatments. This strategic nutritional intervention ensured that the dietary formulations were isonitrogenous and isocaloric, thereby facilitating direct comparison of the effects attributable solely to the source of the meal rather than differences in overall nutrient content.

The gut microbiota and its fermentation metabolites are increasingly recognized for their pivotal role in modulating human and animal health, acting as key players in nutrition, metabolism, and immune function [34]. In the present study, compared with the corn–soybean meal, miscellaneous meals (rapeseed meal, cottonseed meal, and sunflower meal) replacing soybean meal partly and entirely in feed had no significant effects on the relative abundance, the alpha-diversity, or the beta-diversity of fecal microbiota. This is the same as our results in 50–75 kg growing pigs and finishing pigs [13,35]. Previous studies have established that *Firmicutes* and *Bacteroidetes* are the two most abundant bacterial phyla in pigs [36]. Furthermore, a study suggests that rapeseed meal has potential protection effects on the immunological homeostasis of pigs, maybe via modulation of gut microbiome functions [37]. In an experiment with nursery pigs, supplementing the diet with 20% rapeseed meal significantly lowered the relative abundance of *Bacteroidetes* while generally increasing that of *Firmicutes* [38]. Shuai et al. also found that fermented rapeseed meal significantly decreased the abundance of Escherichia coli, but increased Lactobacillus in the cecum of growing pigs [39]. Fermented cottonseed meal replacing soybean meal enhanced Lactobacillus in the ileum and Muribaculaceae in the cecum of weaned piglets [22]. In contrast to these findings, our study indicates that miscellaneous meals (rapeseed meal, cottonseed meal, and sunflower meal) replacing soybean meal partly and entirely in the diet has no significant effects on the microbiota composition in the feces of pigs during the 25–50 kg stage. However, there is currently little research on the microbiota of developing pigs fed rapeseed meal, cottonseed meal, or sunflower meal rather than soybean meal, necessitating more investigation.

SCFAs, produced by gut bacteria by fermenting non-digestible dietary fiber, can promote the absorption of water and electrolytes in the colon, maintain the integrity of the intestinal barrier, and relieve diarrhea [40,41]. SCFAs are regarded as important mediators in the communication between the intestinal microbiome and the immune system [42]. SCFAs are important metabolites for maintaining intestinal homeostasis [43]. Previous studies have shown that adding short-chain fatty acids can promote energy metabolism and growth performance in mice [44,45]. Our results show that compared with the soybean meal diet, miscellaneous meals that replace soybean meal partly significantly increased butyric acid and valeric acid contents in feces. Butyric acid, produced mainly by the gut microbial fermentation of unabsorbed carbohydrates and dietary fiber in the cecum and colon, is approximately 20% of the total short-chain fatty acids [46,47]. Valeric acid is produced mainly by fermentation of proteins and amino acids [47]. Butyrate is preferentially absorbed by colon epithelial cells as an energy source, thereby promoting epithelial cell proliferation and damage repair [44,48]. Lu et al. found that adding 500 mg/d of butyric acid orally from the fourth day after birth to weaning can significantly increase daily weight gain by 13% in piglets [49]. A previous study also showed that butyrate might protect epithelial cells from LPS-induced impairment of barrier integrity through an increase in the synthesis of tight junction proteins, and perhaps regulation of energy homeostasis [50]. On the other hand, our results also show that compared with the soybean meal diet, miscellaneous meals replacing soybean meal entirely, significantly reduce acetic acid, propionic acid, and isobutyric acid contents in feces. Feed-fermented rapeseed meal had higher butyrate content in the cecum than the pigs with a corn–soybean meal diet [39]. In our study, the content of SCFAs may be associated with the usage of miscellaneous meals. Excessive miscellaneous meals can reduce the SCFAs content in growing pigs during the 25–50 kg stage.

## 5. Conclusions

In conclusion, the results of the present study indicate that miscellaneous meals (rapeseed meal, cottonseed meal, and sunflower meal) replacing soybean meal partly or entirely, significantly improve the growth performance of growing pigs during the 25–50 kg stage. Replacing soybean meal entirely also improved serum contents of TP, HDL-C, LDL-C, and serum free amino acid content. Partially replacing soybean meal increased butyric acid and valeric acid contents in feces. In addition, miscellaneous meals replacing soybean meal partly or entirely had no negative effects on the apparent total tract digestibility of nutrients and fecal microbiota composition. Therefore, it is demonstrated that miscellaneous meals (rapeseed meal, cottonseed meal, and sunflower meal) could replace soybean meal partly as a basal diet in growing pigs during the 25–50 kg stage. Thus, this study revealed that miscellaneous meals are a functional alternative feed ingredient to soybean meal in pig diets.

## Figures and Tables

**Table 1:** Ingredient composition of experimental diets in growing pigs.

Ingredients%	CON	CSM	CMM	Calculated Nutrient Levels	CON	CSM	CMM
Corn	73.07	69.91	66.93	Digestive energy (Kcal/kg)	3397	3395	3392
Soybean meal	22.10	11.00		Metabolic energy (Kcal/kg)	3310	3296	3284
Rapeseed meal		4.50	8.50	Net energy (Kcal/kg)	2475	2475	2475
Cottonseed meal		3.98	8.62	CP, %	16	16	16
Sunflower seed meals		4.50	8.50	EE, %	3.47	4.86	6.20
Soybean oil	0.59	1.78	2.90	CF, %	2.31	3.67	4.99
Limestone	0.74	0.87	1.01	ADF, %	3.27	4.49	5.71
Dicalcium phosphate	1.29	0.97	0.67	NDF, %	8.47	10.65	12.76
NaCl	0.40	0.40	0.40	Ash, %	4.76	4.68	4.61
L-lysine sulfate	0.35	0.52	0.70	Ca, %	0.66	0.66	0.66
DL-Met	0.05	0.04	0.04	Total P, %	0.56	0.56	0.56
L-Thr	0.09	0.14	0.19	STTD P, %	0.40	0.36	0.31
L-Trp	0.01	0.03	0.05	Total Lysine, %	1.10	1.10	1.10
L-Val		0.04	0.09	SID Lysine, %	0.98	0.98	0.98
L-Ile		0.01	0.09	SID Met, %	0.28	0.28	0.28
Choline chloride(50%)	0.10	0.10	0.10	SID Thr, %	0.59	0.59	0.59
Titanium dioxide	0.40	0.40	0.40	SID Trp, %	0.17	0.17	0.17
Vitamin–mineral premix ^1^	0.81	0.81	0.81	SID Val, %	0.64	0.64	0.64
Total	100.00	100.00	100.00	SID Ile, %	0.58	0.58	0.58
				Analyzed nutrient levels			
				CP, %	16.48	16.48	16.47
				EE, %	3.94	5.54	6.79
				Gross energy	3821	3838	3836

^1^ The premix provided the following per kg of diets: VA 4 500 IU, VD2 100 IU, VE 22.5 mg, VK 3.75 mg, VB1 2.25 mg, VB2 7.5 mg, nicotinic acid 30 mg, D-pantothenic acid 11.25 mg, folic acid 0.75 mg, VB6 3 mg, VB12 0.03 mg, biotin 0.08 mg, Fe(FeSO_4_·H_2_O)112.5 mg, Cu(CuSO_4_·5H_2_O)6 mg, Mn(MnSO_4_·H_2_O)4.5 mg, Zn(ZnSO_4_·H_2_O)60 mg, I(CaI_2_O_6_)0.14 mg, and Se(Na_2_SeO_3_)0.3 mg. Abbreviations: CON, corn–soybean meal; CSM, corn–soybean–miscellaneous meal; CMM, corn–miscellaneous meals; SID, standardized ileal digestible; CP, crude protein; EE, ether extract; CF, crude fiber; ADF, acid detergent fiber; NDF, neutral detergent fiber; Ash, crude ash; Ca, calcium; P, phosphorus; STTD, standardized total tract digestibility.

**Table 2:** The measured nutrient values in rapeseed meal, cottonseed meal, and sunflower seed meal.

Items	Crude Protein %	Ether Extract %	Crude Fiber %	Ca %	STTD P %
Rapeseed meal	38.6	1.4	11.8	0.65	0.25
Cottonseed meal	47.0	0.5	10.2	0.25	0.28
Sunflower seed meal	36.5	1.0	10.5	0.27	0.29

**Table 3:** The effect of miscellaneous meals replacing soybean meal partly or entirely in feed on growth performance of 25–50 kg growing pigs ^1^.

Items	CON	CSM	CMM	*p*-Value
IBW (kg)	25.8 ± 0.24	25.78 ± 0.22	25.79 ± 0.21	0.992
FBW (kg)	49.42 ± 0.86	50.29 ± 0.53	50.83 ± 0.73	0.397
ADG (kg/d)	0.89 ± 0.02 ^b^	0.94 ± 0.02 ^a^	0.96 ± 0.02 ^a^	0.028
ADFI (kg/d)	1.68 ± 0.04	1.73 ± 0.04	1.76 ± 0.03	0.365
F/G	1.89 ± 0.03	1.83 ± 0.02	1.83 ± 0.02	0.098

^1^ Values are means and standard error of the means (n = 6). ^a,b^ Means in the same row with different superscripts differ (*p* < 0.05). Abbreviations: IBW, initial body weight; FBW, final body weight; ADG, average daily gain; ADFI, average daily feed intake; F/G, feed to gain ratio. CON, corn–soybean meal; CSM, corn–soybean–miscellaneous meal; CMM, corn–miscellaneous meal.

**Table 4:** The effect of miscellaneous meals replacing soybean meal partly or entirely in feed on apparent digestibility of nutrients in 25–50 kg growing pigs ^1^.

Items	CON	CSM	CMM	*p*-Value
Crude protein (%)	76.56 ± 1.14	77.80 ± 0.76	73.88 ± 1.34	0.067
Ether extract (%)	77.24 ± 1.33	79.78 ± 0.50	78.55 ± 0.93	0.218
Gross energy (%)	82.92 ± 0.70 ^a^	82.73 ± 0.47 ^a^	78.45 ± 0.54 ^b^	<0.001

^1^ Values are means and standard error of the means (n = 6). ^a,b^ Means in the same row with different superscripts differ (*p* < 0.05). Abbreviations: CON, corn–soybean meal; CSM, corn–soybean–miscellaneous meal; CMM, corn–miscellaneous meal.

**Table 5:** The effect of miscellaneous meals replacing soybean meal partly or entirely in feed on serum biochemical parameters of 25–50 kg growing pigs ^1^.

Items	CON	CSM	CMM	SEM	*p*-Value
TP (g/L)	58.94 ^b^	62.77 ^a^	63.88 ^a^	0.78	0.014
ALT (U/L)	56.57	50.09	59.27	3.28	0.530
CRE (umol/L)	112.66	108.84	112.63	2.58	0.806
AST (U/L)	37.44	33.67	38.86	1.98	0.572
ALP (U/L)	217.61	190.83	208.99	7.07	0.304
ALB (g/L)	4.12	4.09	7.31	1.08	0.401
UREA (mmol/L)	3.08	2.47	3.33	0.19	0.182
GLU (mmol/L)	3.45	3.05	3.46	0.28	0.815
TG (mmol/L)	0.43	0.43	0.30	0.05	0.428
CHO (mmol/L)	0.86	0.83	0.87	0.02	0.750
HDL-C (mmol/L)	0.89 ^b^	0.83 ^b^	1.01 ^a^	0.03	0.005
LDL-C (mmol/L)	1.51 ^b^	1.51 ^b^	1.78 ^a^	0.05	0.046

^1^ Values are means and standard error of the means (n = 6). ^a,b^ Means in the same row with different superscripts differ (*p* < 0.05). The overall *p*-values obtained by one-way ANOVA analysis for the six treatment groups. Abbreviations: TP, total protein; CRE, creatinine; AST, aspartate aminotransferase; ALP, alkaline phosphatase; ALT, alanine aminotransferase; ALB, albumin; UREA, urea; GLU, glucose; TG, triglyceride; CHO, cholesterol; HDL-C, high-density lipoprotein cholesterol; LDL-C, low-density lipoprotein cholesterol; CON, corn–soybean meal; CSM, corn–soybean–miscellaneous meal; CMM, corn–miscellaneous meal.

**Table 6:** The effect of miscellaneous meals replacing soybean meal partly or entirely in feed on serum free amino acid content in 25–50 kg growing pigs ^1^.

Items	CON	CSM	CMM	SEM	*p*-Value
Essential amino acids					
Lys	139.28 ^b^	193.60 ^b^	299.07 ^a^	23.2	0.007
Met	23.59	22.25	35.76	2.84	0.189
Thr	103.13 ^b^	111.93 ^b^	180.68 ^a^	14.14	0.038
Val	170.72 ^b^	216.26 ^b^	349.05 ^a^	21.15	<0.001
Ile	76.81 ^b^	92.71 ^b^	134.13 ^a^	8.53	0.008
Leu	157.24 ^b^	184.23 ^b^	244.23 ^a^	11.76	0.002
Phe	60.84 ^b^	67.98 ^b^	101.18 ^a^	5.05	<0.001
His	31.04	30.95	45.93	3.43	0.118
Arg	82.96 ^b^	109.6 ^b^	173.83 ^a^	14.67	0.022
Non-essential amino acids					
Ser	93.97	89.03	97.29	3.84	0.703
Ala	526.8	371.88	406.71	28.81	0.061
Gly	1135.85 ^a^	1010.9 ^ab^	799.27 ^b^	58.13	0.046
Tyr	66.07	62.16	85.48	4.34	0.053
Glu	221.88	207.11	187.17	13.90	0.621
Asp	12.67	12.42	11.2	0.86	0.777
Cys	32.54 ^b^	30.06 ^b^	38.94 ^a^	1.32	0.008
Hypro	121.33	110.67	113.33	5.49	0.736
Pro	190	175.33	212	10.11	0.350
Non-protein amino acids and derivatives/metabolites of amino acids					
Tau	128.76	135.93	150.85	8.06	0.550
Urea	2225.27	2140.48	2756.45	159.34	0.242
α-AAA	46.30	43.87	77.35	8.95	0.246
Cit	49.97 ^b^	58.13 ^ab^	67.75 ^a^	2.78	0.021
Orn	72.95	54.72	94.40	9.14	0.215

^1^ Values are means and standard error of the means (n = 6). ^a,b^ Means in the same row with different superscripts differ (*p* < 0.05). Abbreviations: CON, corn–soybean meal; CSM, corn–soybean–miscellaneous meal; CMM, corn–miscellaneous meal; Lys, lysine; Met, methionine; Thr, threonine; Val, valine; Ile, isoleucine; Leu, leucine; Phe, phenylalanine; His, histidine; Arg, arginine; Ser, serine; Ala, alanine; Gly, glycine; Tyr, tyrosine; Glu, glutamate; Asp, aspartic acid; Cys, cysteine; Hypro, hydroxyproline; Pro, proline; Tau, Taurine; α-AAA, aromatic amino acids; Cit, citrulline; Orn, ornithine.

**Table 7:** Effect of miscellaneous meals replacing soybean meal partly or entirely in feed on SCFA levels in the fecal contents of 25–50 kg growing pigs ^1^.

Items	CON	CSM	CMM	*p*-Value
Acetic acid	30.7 ± 2.96 ^a^	31.5 ± 2.2 ^a^	15.04 ± 1.59 ^b^	<0.001
Propionic acid	21.72 ± 2.41 ^a^	25.89 ± 3.43 ^a^	12.98 ± 1.83 ^b^	0.011
Isobutyric acid	4.2 ± 0.54 ^a^	3.96 ± 0.48 ^a^	2.19 ± 0.26 ^b^	0.009
Butyric acid	5.08 ± 0.78 ^b^	8.2 ± 0.94 ^a^	4.02 ± 0.6 ^b^	0.005
Isovaleric acid	3.95 ± 0.86	4.07 ± 0.54	2.52 ± 0.32	0.137
Valeric acid	2.2 ± 0.38 ^b^	3.35 ± 0.44 ^a^	1.6 ± 0.19 ^b^	0.009

^1^ Values are means and standard error of the means (n = 6). ^a,b^ Means in the same row with different superscripts differ (*p* < 0.05). Abbreviations: CON, corn–soybean meal; CSM, corn–soybean–miscellaneous meal; CMM, corn–miscellaneous meal.

## Data Availability

Data are contained within the article.
